# Comprehensive Characterization of Oligolactide Architecture by Multidimensional Chromatography and Liquid Chromatography–Mass Spectrometry

**DOI:** 10.1021/acsomega.5c08063

**Published:** 2026-01-16

**Authors:** Amit Lal, Paul Severin Eselem Bungu, Karola Luetzow, Sarah Kirchhecker, Regine Apostel, Olaf Lettau, Monique Hannemann, Harald Pasch, Francesca M. Toma

**Affiliations:** † Institute of Functional Materials for Sustainability, Helmholtz-Zentrum Hereon, Kantstr. 55, 14513 Teltow, Germany; ‡ Institute of Active Polymers, Helmholtz-Zentrum Hereon, Kantstr. 55, 14513 Teltow, Germany; § Faculty of Mechanical and Civil Engineering, Helmut-Schmidt-University, 22043 Hamburg, Germany

## Abstract

Oligolactides (OLA) are increasingly finding applications in drug delivery systems, implant coating, and tissue engineering. The most common synthetic route to OLA, ring-opening polymerization, produces polymer chains with complex architecture, including cyclic structures, chains with acid end groups, and initiator-bound oligomeric species. This inherent molecular heterogeneity presents significant challenges in analytical characterization. To address this complexity, it is essential to develop a robust separation method for comprehensive analysis. In this study, we developed a novel and robust high-performance liquid chromatography (HPLC) method using ethanol-modified chloroform as an eluent on a normal-phase column, which effectively separates the OLA chains. Additionally, the impact of different initiators on the molecular heterogeneity of OLA chains was investigated. To elucidate the separation mechanism, the HPLC method is hyphenated with size exclusion chromatography in a two-dimensional liquid chromatography setup. The results indicate that OLA chains are separated based on increasing degree of polymerization or increasing lactide incorporation. Furthermore, online coupling of HPLC with mass spectrometry provided deeper insight into the heterogeneity of the bulk. This study highlights the importance of correlative characterization techniques as a preferred approach for determining microstructural heterogeneity in low molar mass OLA samples.

## Graphical abstract

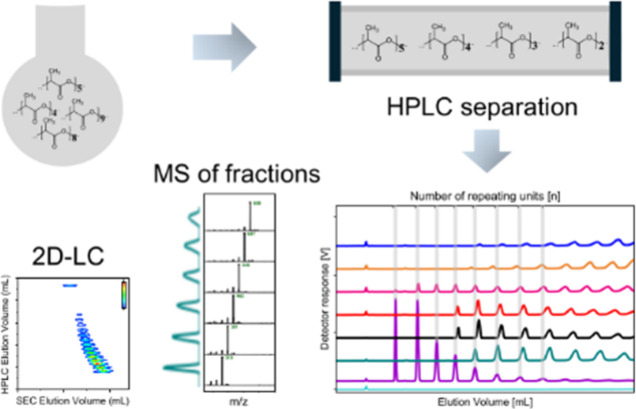

## Introduction

Polylactic acid (PLA), an aliphatic polyester derived from biobased feedstocks such as cornstarch and sugar cane, shows mechanical properties comparable to petrochemically produced plastics.^1^ Life cycle assessments indicate that PLA sequesters substantial amounts of carbon dioxide, making it a preferred choice for industries transitioning toward sustainability and incorporating it into more durable, long-term products.^2^ PLA is widely used in packaging, agriculture, consumer goods, and healthcare. Its biodegradability and biocompatibility makes it particularly relevant for biomedical applications, including implant and drug delivery systems.^3^

While PLA has been extensively characterized, its oligomeric esters have received comparatively less attention. However, the growing use of well-defined OLAs in biomedical applications^4–6^ presents a significant challenge in managing heterogeneity for quality control in material design. The chemical heterogeneity of OLA primarily arises from its synthesis process. Commercially, OLA is produced via the ring-opening polymerization (ROP) of lactide, a cyclic dimer of lactic acid. This polymerization is typically catalyzed by tin­(II) octanoate (Sn­(Oct)_2_), which is first activated by an alcohol or another hydroxyl-containing molecule. The reaction proceeds through nucleophilic attack by the activated catalyst on the lactide monomer, followed by coordination–insertion, yielding polymer chains with ester end groups (-OR), where R originates from the initiator.^7^

However, in the presence of moisture as dictated in Scheme 1, water can activate the catalyst, leading to side reactions that generate OLA chains with acid (COOH) end groups.^8^ Additionally, secondary reactions such as cyclization, racemization, and transesterification can alter the stereochemistry and sequence structure of the oligomeric subunits throughout the polymerization process^9–11^ . As a result, OLAs exhibit microstructural heterogeneity, with variations in functionality, end groups, stereochemistry, leading to a complex molecular architecture as shown in Scheme 1.

**Scheme 1:**
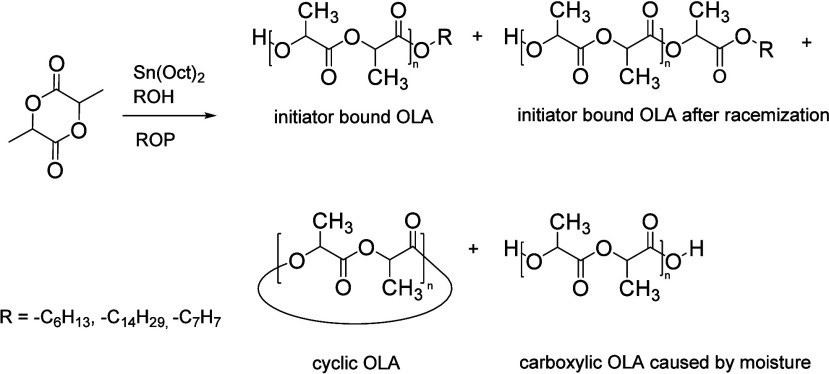
Synthesis of OLA by ROP and possible reactions products

Given the challenges in controlling monomer sequence and chain length in oligomers,^12^ developing precise analytical methods to examine their microstructure is essential. A detailed investigation of OLAs microstructural heterogeneity not only provides insights into synthetic pathways but also enables early detection of undesired byproducts during polymerization, ultimately improving material design.

Since the microstructural heterogeneity is multidimensional, a single analytical technique is insufficient to resolve the overlapping distributions. To effectively analyze these complex, multivariate distributions, a combination of complementary analytical techniques is required. Spectroscopic methods such as nuclear magnetic resonance (NMR) and Fourier transform infrared (FTIR) spectroscopy are valuable for functional and end group characterization in polymer analysis. However, these techniques primarily provide average structural information and often lack the resolution needed to distinguish molecular composition distributions in detail.^13^ Size exclusion chromatography (SEC) is commonly used to separate molecules based on hydrodynamic radius, but it has limitations in correlating size-based distribution with molecular structure. Since SEC relies on size exclusion principles, its effectiveness is enhanced when combined with molar mass-sensitive detectors such as multiangle light scattering (MALS) and online viscometers.

High performance liquid chromatography (HPLC) is the most widely used technique for oligomer characterization, offering the ability to separate oligomers based on both the number of repeating monomer units and functionality type distribution. Its robust separation capabilities combined with sensitive detection makes it suitable for a broad range of molecules regardless of molar mass or volatility. Advances in HPLC method include the integration of a second dimension to simultaneously resolve interrelated distributions.^13^ This can be achieved by the coupling of HPLC with other chromatographic techniques, such as SEC in a two-dimensional liquid chromatography (2D-LC) setup, or by hyphenating HPLC with structure-sensitive detectors such as NMR, FTIR, MS either online or offline. These advancements further enhance the role of HPLC in elucidating complex molecular distributions in oligomers.

The separation of lactic acid (LA) and its oligomers using aqueous systems dates to 1952, when Montgomery employed a silicic acid column with a chloroform-butanol mobile phase to separate monomers, dimers, and trimers.^14^ In 2005, Vu et al. used gradient elution on a C18 column with water and acetonitrile as eluents to separate short lactide chains.^15^ Codari et al. improved this method by broadening its applicability to a wider range of low molar mass PLA, employing a 30 min gradient elution on a C18 column with water/acetonitrile acidified with 0.1 vol% phosphoric acid.^16^ Li et al. applied normal-phase chromatography to separate PLA based on stereochemistry.^17^ Radke et al. used liquid chromatography at critical conditions (LCCC) to distinguish between linear and star-shaped PLA.^18^ Falkenhagen et al. used a gradient elution LC to determine the degree of functionalization of star-shaped polylactides modified by acetylation.^19^ Recently, Capecchi et al. developed a normal phase interaction chromatographic (IC) method with mass spectrometry for OLA impurity quantification on a silica column with hexane/ethyl acetate mobile phase, though with poor resolution.^20^

Bungu et al. refined Li’s method for separating PLA molecules by focusing on stereochemical heterogeneity.^21^ They optimized the eluent composition to achieve more precise separation of PLA chains with slight variations in d-lactide content. This enhanced method improved the separation of PLA chains with subtle stereochemical differences, offering greater control over polymer microstructure.

The present study aims to develop and validate a robust normal-phase HPLC method adopted from the Bungu approach^21^ for selectively separating OLA chains. Selected standards will guide method development, which will then be validated using lab-synthesized samples. Investigating OLA chains initiated by different initiators will provide insight into their influence on chemical heterogeneity during the early stages of polymerization. This understanding can enhance synthetic efficiency and provide valuable information on reaction kinetics. Developing a reliable separation method will facilitate the analysis of oligomers across a broad molar mass range. Online coupling of this HPLC method with SEC in a 2D-LC setup will provide a bivariate distribution of molar mass and chemical composition, confirming the separation mechanism. Additionally, MALDI-TOF MS offers a closer examination of the heterogeneity in sample composition, while integrating the LC method with mass spectrometry will enable comprehensive characterization of the sample’s end-group functionality distribution. This approach allows for a detailed analysis of OLA products, capturing both molar mass and functionality type distributions.

## Experimental Section

### Chemicals

For HPLC analysis, chloroform (TCM) and ethanol were used as solvents, purchased from J.T. Baker and Sigma-Aldrich, respectively. Both solvents were of HPLC grade and used without further purification. OLA samples were synthesized by ROP of l-lactide catalyzed by Sn­(Oct)_2_ and using the initiators 1-hexanol, 1-tetradecanol, and benzyl alcohol (experimental details can be found in the Supporting Information). PLA1.5K, a commercial GPC standard, was purchased from PSS Polymer Standards Service GmbH, now a part of Agilent Technologies (Mainz, Germany). The molecular parameters of all samples are listed in Table 1.

**Table 1:** Molar mass data for the synthesized samples were obtained by SEC using universal calibration^a^

Sl. No.	Sample name	*M*_n_ [g·mol^–1^]	*M*_w_ [g·mol^–1^]	Dispersity	Initiator
1	OLATD0.7	730	1087	1.4	tetradecanol
2	OLATD1.3	1285	1744	1.3	
3	PLA1.5K	971	1411	1.4	
4	OLABA0.7	741	786	1.1	benzyl alcohol
5	OLABA1.3	1290	1395	1.1	
6	OLAHX0.8	835	1373	1.6	hexanol
7	OLAHX1.0	1084	1723	1.6	
8	OLAHX1.5	1570	1875	1.2	

a Three sets of samples were synthesized using tetradecanol, hexanol, and benzyl alcohol as initiators. The sample nomenclature corresponds to laboratory designations.

### Chromatographic Instrumentation

All instruments, software, calibration standards and columns are from Agilent Technologies (Santa Clara, CA, USA) or PSS Polymer Standards Service GmbH (Mainz, Germany), now a part of Agilent Technologies, unless stated otherwise.

### Interaction Chromatography (IC)

The liquid chromatography (LC) system used in this study was an Agilent 1200 series LC system, equipped with a quaternary pump, an autosampler, a degasser, column heating compartment, and a UV detector. Additionally, an Evaporative Light Scattering Detector (ELSD) 1260 Infinity II was connected for detection. Separation was performed using a Nucleosil 100 Å silica column (5 μm, 250 mm length, and 4.6 mm ID) from Macherey Nagel GmbH and Co. KG (Düren, Germany). Data acquisition and processing were performed using WINGPC UniChrom Build 9050 Software. Prior to injection, all samples were dissolved in the mobile phase at a concentration of 1 mg·mL^–1^ and 30 μL was injected during analysis. The applied gradient for the separation is shown in Figure 2.

### Size Exclusion Chromatography (SEC)

Molar mass and dispersity measurements were performed using an Agilent 1260 Infinity II GPC system, equipped with an isocratic pump, degasser, autosampler, column-heating compartment, UV detector, and refractive index (RI) detector. Additionally, a PSS SLD7000 Multi-Angle Light Scattering (MALS) detector and a PSS DVD1260 online viscometer were used to determine absolute molar masses based on light scattering and universal calibration. Polymer separation was achieved using a Lux guard column (10 μm, 50 mm × 8 mm ID) and two SDV analytical, linear XL columns (10 μm, 300 mm × 8.0 mm ID). Molar mass evaluation was conducted using WinGPC UniChrom software (version 8.3 Build 9050) with a universal calibration approach based on polystyrene standards. (*M*_n_ ranging between 580 g·mol^–1^ and 975 000 g·mol^–1^). The mobile phase consisted of TCM stabilized with ethanol (0.6–1 vol%) at a flow of 1 mL·min^–1^. Samples (4 mg) were dissolved in 2 mL containing toluene (0.1 vol%) and left to dissolve overnight. A 50 μL aliquot of the solution was injected, with the toluene peak serving as the flow rate maker. Molar masses were determined via SEC using intrinsic viscosity and universal calibration, eliminating the need for prior d*n*/d*c* determination. All measurements were performed in triplicate.

### Two-Dimensional Liquid Chromatography (2D-LC)

Two-dimensional chromatography separations were performed by coupling the IC and SEC using a valve system (VICI Valco instrument, Houston, Texas, USA). The system was controlled by the WinGPC UniChrom software (version 8.4 Build 9999) and equipped with two 100 μL sample loops. Samples (4 mg) were dissolved in TCM (1 mL), and a 100 μL solution was injected into the first-dimension column. The first-dimension separation was conducted using an Agilent 1260 infinity II LC system equipped with a quaternary pump, degasser, autosampler, column heating compartment, and a UV detector. Fractionation was achieved using a Nucleosil–OH column (4.6 mm × 250 mm and particle size of 5 μm) from Macherey-Nagel GmbH and Co. KG (Düren, Germany) under controlled flow conditions. The mobile phase gradient detailed in Figure S7, was applied at a flow rate of 0.05 mL·min^–1^. The second-dimension separation was performed using a PSS SECcurity 2 SEC system, equipped with an isocratic pump, degasser, autosampler, column-heating compartment, and UV detector. The SEC system further separated the HPLC fractions based on their size (molar mass) using a PL Rapide M column (7.5 mm × 150 mm and particle size of 3 μm) at a flow rate of 2.75 mL·min^–1^. Data were collected using an Agilent 1290 infinity II ELSD (G7102A) and processed using WinGPC UniChrom software (version 8.4, Build 9999).

### Liquid Chromatography Coupled to Mass Spectrometry

For LC-MS analysis, analytes (4 mg) were dissolved in TCM (1 mL), and a 100 μL aliquot was injected into the Agilent 1260 Infinity II HPLC system. The system was equipped with a quaternary pump, degasser, autosampler, column heating compartment, and UV detector. The separated analytes were subsequently directed to an Agilent InfinityLab LC/MSD XT mass spectrometer for detection. Data analysis was performed using OpenLab CDS software. Atmospheric Pressure Chemical Ionization (APCI) served as the chosen ionization source due to its suitability for analyzing small, heat-stable molecules commonly found in OLA samples. Mass spectrometer conditions included tailored fragmentor voltages of 150 V for low molar mass and 350 V for high molar mass species, positive polarity mode, a mass range of 100–2000 *m*/*z*, a capillary voltage of 3000 V, and corona currents of 10 μA. These settings optimized sensitivity, resolution, and accuracy, facilitating detailed characterization of OLA samples.

### Matrix-Assisted Laser Desorption Ionization Time-of-Flight Mass Spectrometry (MALDI-TOF-MS)

MALDI-TOF mass spectra were recorded on a Bruker Ultraflex instrument (Bruker Daltonics, Bremen, Germany) in both linear and reflective modes. Samples (10 mg·mL^–1^), the matrix compound trans-2-[3-(4-*tert*-butylphenyl)-2-methyl-2-propenylidene] malononitrile (DCTB, 20 mg·mL^–1^), and the ionizing agent sodium trifluoroacetate (NaTFA,13 mg·mL^–1^) were dissolved in tetrahydrofuran (THF) and mixed in a 3:20:1 ratio. A 1 μL aliquot of this mixture was applied to designated spots on a MALDI ground steel target plate. Spectra acquisition and analysis were conducted using FlexControl, FlexAnalysis, and PolyTools software (Bruker Daltonics). Typically, 1000 laser shots were accumulated from different positions on the sample spot. Calibration was performed using PMMA 1.6k (Polymer Standards Service GmbH, Mainz, Germany).

## Results

All analyses were conducted using seven OLA samples synthesized using tetradecanol, hexanol, and benzyl alcohol as initiators. The selected samples covered a number-average molar mass (*M*_n_) ranging from 750 g·mol^–1^ to 1570 g·mol^–1^, providing a comprehensive data set for method validation. The synthesized OLAs were characterized by MALDI-TOF mass spectrometry. Figure 1 presents the MALDI-TOF mass spectra of three selected samples synthesized with the listed initiators. While MALDI is well suited for high molar mass samples, the signals below 1000 Da can interfere with the signals of the used matrix, rendering three out of the seven samples unsuitable for MALDI analysis.

**Figure 1:**
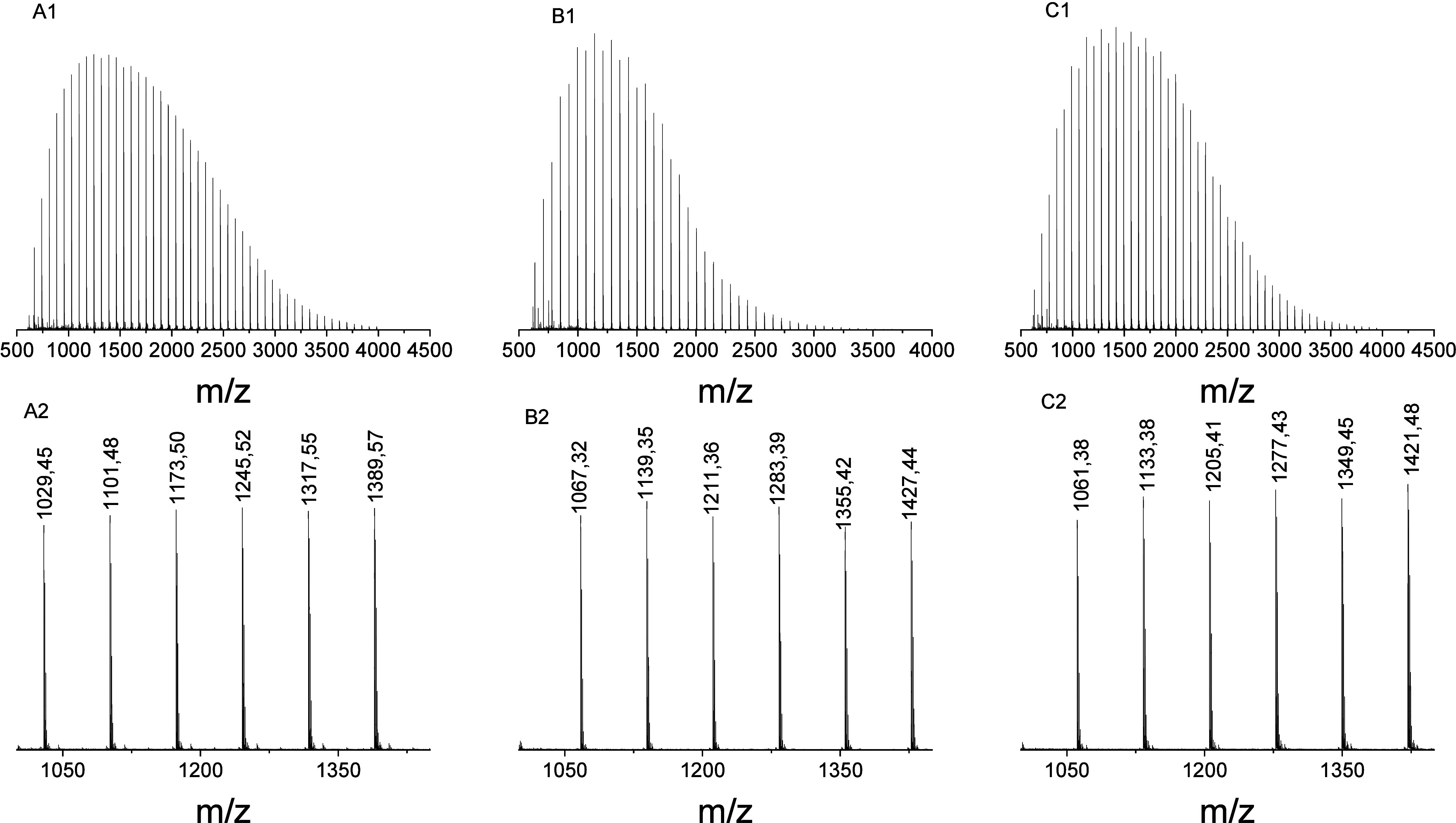
MALDI-TOF MS spectra and enlarged plots of samples (A1) tetradecanol-initiated sample OLATD1.3 and its enlarged plot (A2), (B1) Benzyl alcohol-initiated sample OLABA1.3 and its enlarged plot (B2), and (C1) hexanol-initiated sample OLAHX1.5 and its enlarged plot (C2).

MALDI-TOF mass spectrometry has been widely applied for bulk characterization of PLA, allowing detailed analysis of molecular weight distribution, chain architecture, and end-group composition. Previous studies have demonstrated that functional initiators influence ionization behavior,^22^ tin-catalyzed polymerizations produce mixtures of linear and cyclic chain,^23^ and water-initiated polymerizations yield polymers with distinct acid end groups.^24^ Hence, MALDI-TOF was selected as the initial analytical technique in this work.

MALDI-TOF MS analysis immediately revealed the polydispersity and the heterogeneity of the OLA samples, as indicated by the presence of multiple series and minor peaks. Two distinct series, representing chains with even and odd numbers of lactic acid units, were observed in all samples. Peaks within each series were spaced 144 Da apart, and the odd-numbered series was shifted 72 Da relative to the even-numbered series, indicating transesterification.^25^ For example, the hexanol-initiated OLA (OLAHX1.5) spectrum in Figure 1 **C1** and the expanded region in Figure 1 **C2** display peaks in the *m*/*z* range of 1133.40 to 1421.48, corresponding to OLA chains with lactide repeat units represented by the general formula [H­(C_3_H_4_O_2_)_2n_OC_6_H_13_ + Na]^+^, where n = 7–9. Peaks observed in the *m*/*z* range of 1061.36 to 1349.45 contain an additional lactic acid unit and are represented by [H­(C_3_H_4_O_2_)_2n+1_OC_6_H_13_ + Na]^+^, further confirming the transesterification effect during oligomerization. Similar observations are found for the tetradecanol-initiated and benzyl alcohol-initiated samples (Figures 1A and **1B**).

A closer inspection of the spectrum of tetradecanol initiated samples (OLATD1.3) in Figure 1 **A1** revealed a minor repeating series assigned to cyclic OLAs, described by the general formula [(C_3_H_4_O_2_)_n_ + Na]^+^. This series, identified by analysis software with a relative abundance of approximately 1.4%, exhibited isotopic envelopes (see Figure S8) consistent with the expected mass spacing of OLA species. However, the peaks were of very low intensity and partially merged with the baseline, making reliable quantitative or qualitative analysis challenging. Therefore, the presence of cyclic oligomers is considered tentative. To obtain a more comprehensive view of the sample’s microstructural heterogeneity, the MALDI-TOF MS results were complemented by additional analytical techniques targeting the separation of the different molecules by chemical structure or functionality for end group characterization. OLA chains are moderately hydrophilic due to the presence of hydroxyl (−OH) end groups on ester-bound chains, as well as both carboxyl (−COOH) and (−OH) end groups on acid-bound chains. The separation of OLA chains by chemical structure may be achieved with liquid chromatography using a polar stationary phase, where selective adsorption and desorption occur via nonpolar and polar solvents. During method development, TCM and TCM with 4 vol% ethanol ensured retention and complete elution, respectively, and fine-tuning the ethanol content by ± 0.1 vol% significantly impacted retention, with lower mobile phase polarity increasing retention as highlighted in Figure S3 of the Supporting Information. Optimization is achieved by varying the gradient steepness while balancing resolution and analysis time. This observation is consistent with previous studies.^21^ A slow gradient increases solvent polarity, weakening analyte-column interactions, and enabling efficient elution.

Also, temperature significantly influenced separation, with retention increasing unexpectedly from 30 to 55 °C as shown in Figure S4 of the Supporting Information. This finding contrasts with typical adsorption behavior, where higher temperatures favor desorption via entropy changes, but aligns with findings for amphiphilic polymers.^26^ Here, a 0.1 vol% ethanol increase in the eluent composition had a desorption effect similar to a decrease in the column temperature by 10 °C, enabling precise control of OLA separation.

The optimized method presented in Figure 2A, used a step-gradient elution from 0.4 to 1.4 vol% ethanol over 55 min, with an isocratic step introduce at 0.7 vol% ethanol content or at 25 min for 15 min with the column set at 45 °C to enhance resolution.

**Figure 2:**
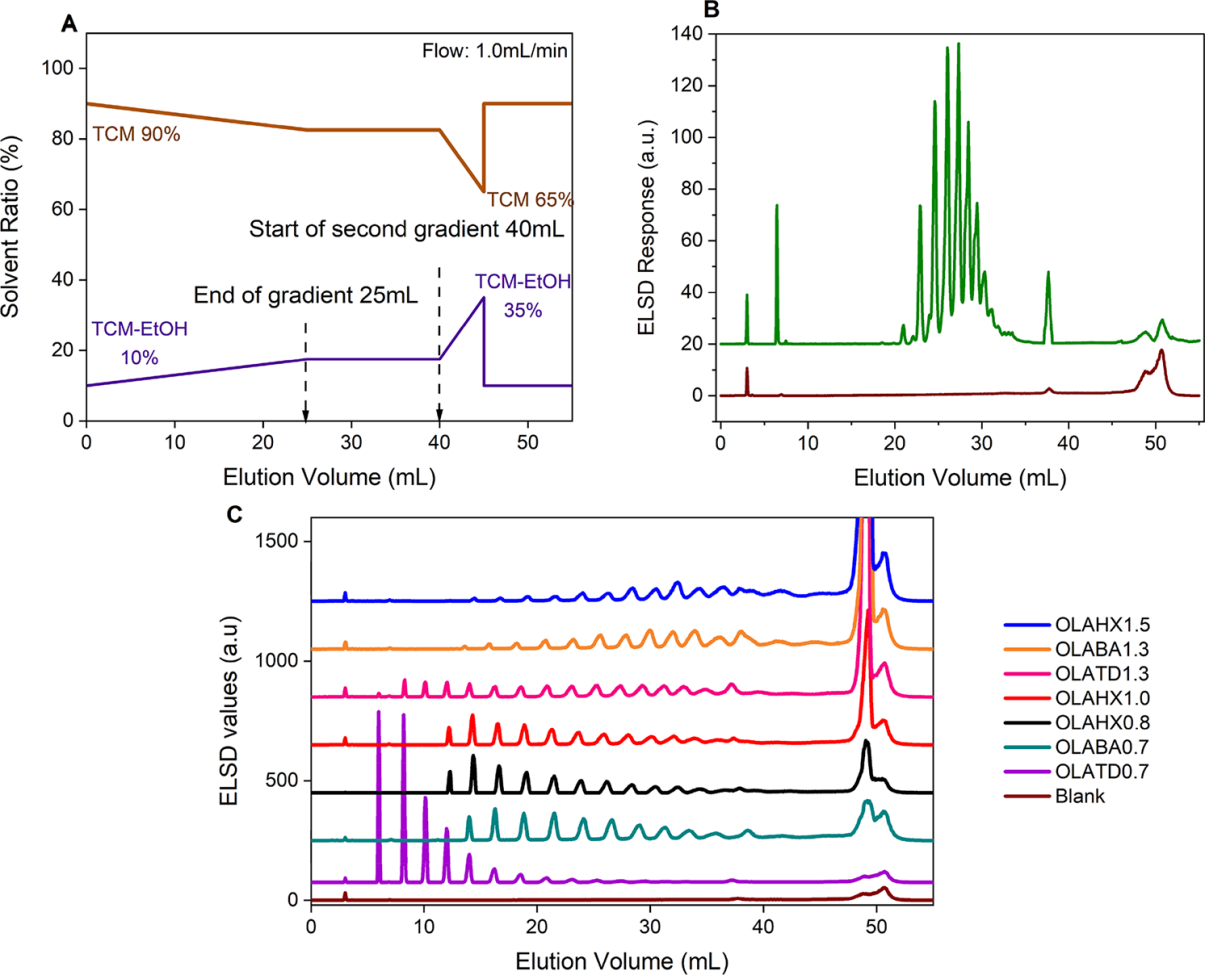
(A) Solvent gradient profile from 0.4 vol% to 1.4 vol% EtOH in TCM (B) Elution profile of PLA1.5K (green), a commercial standard compared to the blank elution (brown), obtained by applied gradient (C). Comparison of elution profiles of OLA samples OLATD0.7 (purple), OLABA0.7­(blue-gree) OLAHX0.8 (black), OLAHX1.0 (red), OLATD1.3 (pink), OLABA1.3 (orange), and OLAHX1.5 (blue) arranged in order of increasing *M*_n_; stationary phase Nucleosil–OH, temperature 45 °C.

Figure 2B shows the application of the method on the PLA1.5K sample, a commercial GPC standard, which served as the basis for method development. The unexpected presence of multiple peaks, in what was assumed to be a homogeneous standard, prompted a more detailed investigation. A total of 16 peaks were observed in the sample but were absent in the blank, confirming heterogeneity within the standard OLA sample. The presence of these peaks suggests the existence of different OLA species within the standard, contributing to its heterogeneity. Additionally, a peak at 49 min was noted, corresponding to the elution of unresolved species at the maximum polarity of the mobile phase, further highlighting the complexity of the standard.

Our method was validated with seven OLA samples showing excellent resolution and minimal solvent interference as shown in Figure 2C. These samples exhibit *M*_n_ between 750 and 1570 g/mol, and were synthesized using tetradecanol, hexanol, and benzyl alcohol initiators. As clearly seen, lower *M*_n_ samples eluted earlier with superior resolution, but baseline separation was lost at *M*_n_ above 1500 g/mol. Further improvements for higher *M*_n_ samples (above 1500 kg/mol) compromised resolution for lower *M*_n_ samples. In all seven samples, the unresolved peak at 49 min can be observed, with greater intensity in higher molar mass samples.

The overlaid elugrams in Figure 3 present samples produced with the same initiator and compare OLA elution behavior across samples with different degrees of polymerization. In Figure 3 **A1**, tetradecanol-initiated samples (OLATD0.7 and OLATD1.3) are compared, showing multiple peaks between 6 and 45 mL. OLATD0.7 exhibits high intensity early eluting peaks that decline in strength with increasing elution volume. On the other hand, OLATD1.3 shows the opposite trend, with lower early eluting peak strength that increases in intensity with higher molar mass, correlating elution volume with molar mass. The enlarged plots in Figures 3 **A2 – C2** reveal early elution peaks around 3–10 mL, unique to tetradecanol-initiated samples. These peaks, absent in hexanol- and benzyl alcohol-initiated ones, suggest compositional differences.

**Figure 3:**
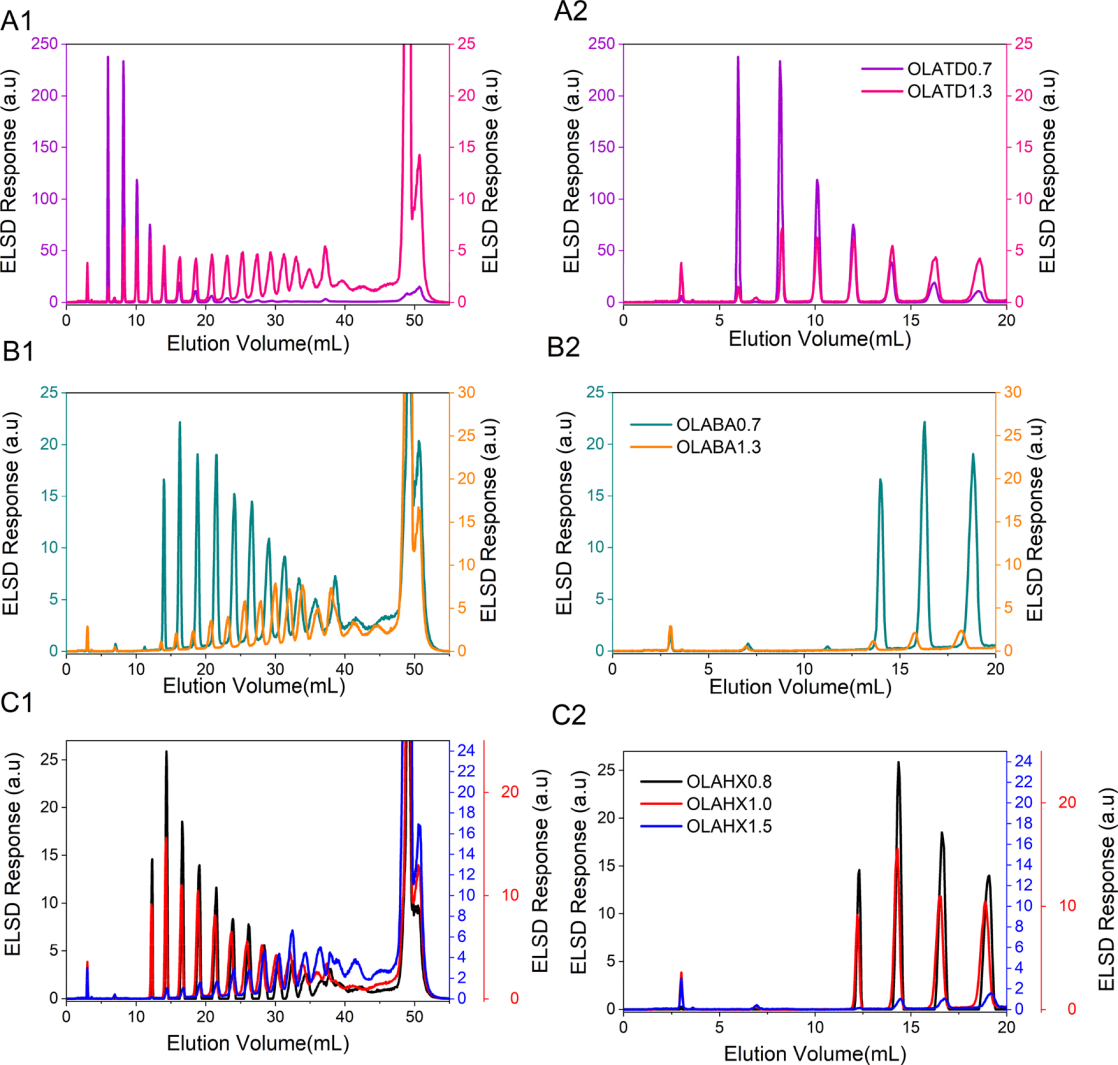
Overlay of elution profiles of (A) tetradecanol-initiated samples OLATD0.7 (purple) and OLATD1.3 (pink), (B) benzyl alcohol-initiated samples OLABA0.7 (blue-green) and OLABA1.3 (orange), and (C) hexanol-initiated samples OLAHX0.8 (black), OLAHX1.0 (red), and OLAHX1.5 (blue) obtained by final method step gradient elution from 0.4 vol% to 1.4 vol% EtOH in TCM; stationary phase Nucleosil–OH, temperature 45 °C.

Despite variations in molar masses, the profiles largely overlap. However, for samples initiated by benzyl alcohol, the low molar mass sample elutes slightly later at higher elution volumes than the higher molar mass counterparts, likely due to end-group differences. This observation warrants further investigation, which will be highlighted later.

To gain deeper insight into the elution characteristics of the OLA chains, the influence of molar mass on the retention behavior was investigated using two-dimensional liquid chromatography (2D-LC). In this setup, fractions separated by chemical interaction in the first dimension HPLC were subsequently analyzed by SEC in the second dimension to resolve differences in molecular size (molar mass). To accommodate the high-throughput SEC column, the HPLC flow rate was reduced 10-fold, requiring the collection of multiple sequential sample slices. For optimal compatibility and reduced analysis time, the original 55 min HPLC gradient (Figure 2A) was shortened to 30 min with corresponding adjustments to the column temperature and gradient slope, as shown in Figure S7. The resulting 2D contour plots of OLATD0.7, OLATD1.2, OLAHX0.8, and OLABA0.7 presented in Figure 4 illustrate the relationship between molecular architecture and molar mass. Across all samples, a general decrease in SEC elution volumes with increasing HPLC elution volume was observed, confirming that the HPLC dimension primarily separates by oligomer chain length.

**Figure 4:**
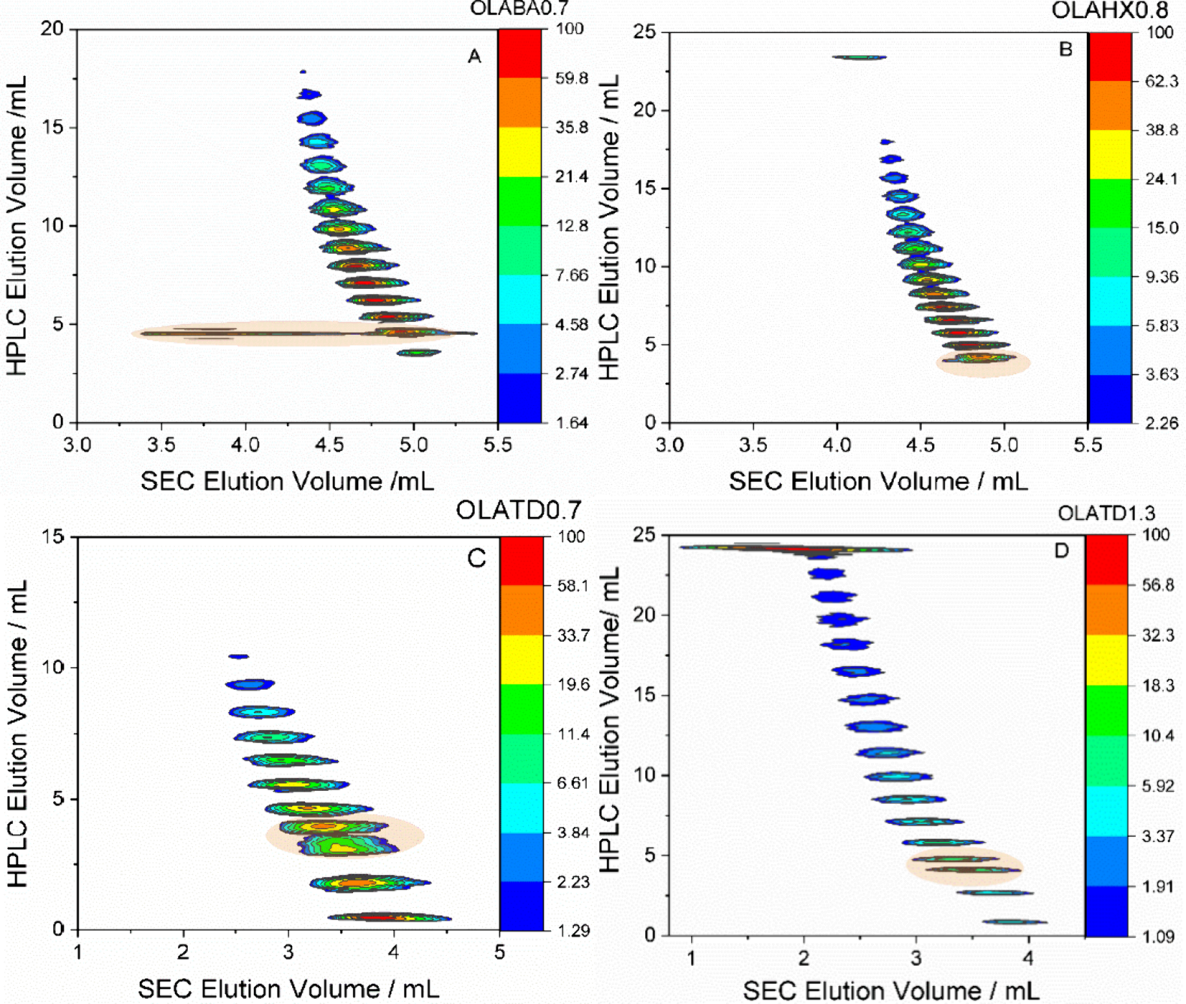
2D-LC contour plots with LC elution vs SEC elution for (A) OLABA0.7, (B) OLAHX0.8, (C) OLATD0.7, and (D) OLATD1.3, using 2D-LC with step gradient elution from 0.5 vol% to 1.4 vol% EtOH in TCM for 30 min described in Figure S7; LC column Nucleosil–OH, SEC column Rapide-M, temperature 30 °C.

Across all samples, a distinctive feature showing two adjacent species coeluting in the HPLC region between 4.2 and 4.65 mL is highlighted in orange. While the C6- and C14-initiated OLAs (OLAHX0.8 and OLATD0.7) display monomeric SEC profile with narrow MMD, consistent with less pronounced molar mass heterogeneity for fractions in this region, the BnOH-initiated sample (OLABA0.7), exhibits bimodal SEC elution with peaks centered at 4.9 and 3.7 mL. This unique bimodal distribution characteristic observed in both the HPLC and SEC dimensions is highlighted in the HPLC and SEC plots in Figure 5A and 5B, respectively and was extracted from the 2D-LC plot of the benzyl alcohol (OLABA0.7) sample. This behavior reflects the coelution of species with differing hydrodynamic volumes suggesting the coexistence of topologically distinct oligomers. Based on this elution behavior, the earlier peak (3.7 mL) likely corresponds to more compact species, possibly cyclic oligomers formed via intramolecular backbiting, whereas the later peak (4.9 mL) may represent linear BnOH-bound chains.

**Figure 5:**
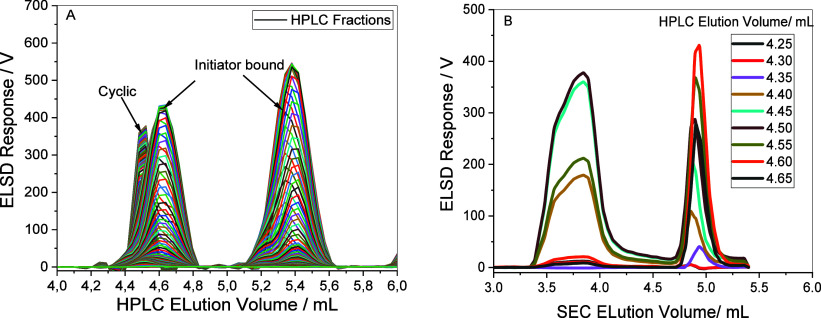
Extracted (A) HPLC and (B) SEC plots from 2D-LC plots of OLABA0.7 display bimodal distributions for HPLC fraction eluting between 4.2 and 4.8 mL.

This observation suggests that while the backbiting reaction occur in all systems, benzyl alcohol initiator system appears to promote propagation of the cyclic species, likely be due to the stabilizing and resonance effect of the benzyl group leading to higher molar masses. To confirm this hypothesis, a more comprehensive characterization where the fractions are isolated and analyzed is imperative.

To further elucidate the molecular characteristics of the coeluting OLA species observed in the 2D-LC experiment, the corresponding HPLC fractions were analyzed using LC-MS. Due to the low polarity of the OLA chains, Atmospheric Pressure Chemical Ionization (APCI), was employed, a soft ionization source, suitable for neutral or weakly polar polymers.

For the hexanol-initiated sample (OLAHX0.8), the mass spectrum reveal two major peak series (Figure 6B). The first series (*m*/*z* ranging from 175.1 to 967.5, Δ_*m*/*z*_ = 72 Da) corresponds to linear OLA chains with hexyl ester end group [H­(C_3_H_4_O_2_)_2n_OC_6_H_13_ + H]^+^ with n = 1–6, along with their transesterification byproduct [H­(C_3_H_4_O_2_)_2n+1_OC_6_H_13_ + H]^+^ with n = 0–5. The second peak series exhibits *m*/*z* 145.05–505.7, which is consistent with cyclic oligomers s [(C_3_H_4_O_2_)_n_+H]^+^ with n = 2–7. Minor peaks corresponding to acid-terminated species [H­(C_3_H_4_O_2_)_n_OH + H]^+^ with n = 1–3 were also identified.

**Figure 6:**
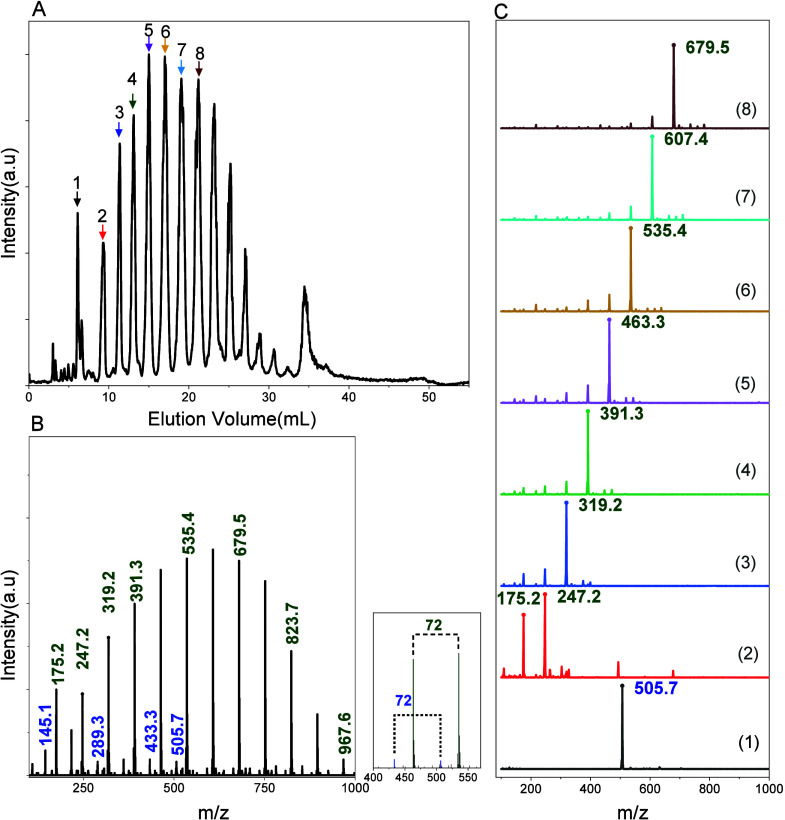
(A) Total ion chromatogram and (B) average spectra MS plot for OLA sample OLAHX0.8 based on final method step gradient from 0.4 to 1.4 vol% EtOH in TCM in 55 min; hexanol initiated OLA series highlighted in green and cyclic OLA species highlighted in blue; (C) individual extracted mass spectra corresponding to peaks of OLAHX0.8 at 6.0,9.3,11.3,13.0, 15.0, 17.0, 19.0, and 21.1 min.

Detailed analysis of the extracted ion chromatograms in Figure 6C revealed a progressive increase in *m*/*z* with increasing elution time (9.3 to 21.1 min), corresponding to linear hexanol-initiated chains [H­(C_3_H_4_O_2_)_n_OC_6_H_1__3_ + H]^+^ with 1 to 8 lactic acid repeat units. This trend confirms that the LC separation primarily depends on oligomers length, with longer chains eluting later.

Interestingly, the first peak in the elugram, eluting at 6.03 min, exhibits a dominant ion at *m*/*z* of 505.4, corresponding to a cyclic OLA species. The extracted mass spectra of the benzyl alcohol-initiated sample (OLABA0.7) likewise shows a coeluting species component consisting of a cyclic species, as illustrated in the extracted mass spectra provided in Figure S10. Although the corresponding fractions display higher molar mass characteristics in the 2D–LC (Figure 5A), their full characterization in the LC-MS was likely limited by reduced ionization efficiency and mass range constraints. The early elution of this species relative to the linear chains is consistent with its compact, ring-like architecture and the absence of polar end groups, which reduces interaction with the stationary phase. Together, these observations support the tentative presence of a cyclic oligomeric fraction, though further detailed investigation is required to confirm its structure and origin.

The observations described above were consistent across OLABA0.7 and OLATD0.7, confirming that LC-MS method effectively separates oligomers based on lactide incorporation. In all three initiator samples, both the cyclic and initiator-bound species were detected, often, coeluted with acid-terminated oligomers. A detailed listing of the identified species is provided in Table 2.

**Table 2:** Identification of OLA Species in Samples with Corresponding Molecular Formulas Based on LC-MS Coupling Using APCI as Ionization Source

initiator	tetradecanol				hexanol				benzyl alcohol		
end group	initiator bound	cyclic	acid bound	end group	initiator bound	cyclic	acid bound	end group	initiator bound	cyclic	acid bound
sample	[M + H]^+^			sample	[M + H]^+^			sample	[M + H]^+^		
OLATD0.7	287.3	145.1		OLAHX0.8	175.1	145	163.1	OLABA0.7	181.1	145.1	
	359.4	217.2			247.2	217	235.1		253.2	217.1	235.1
	431.4	289.3			319.1	289.25	307.2		325.1	289.3	307.2
	503.6	361.1			391.2	361			397.1	361.2	379.1
	575.6	433.4			463.3	433.2			469.3	433.2	
	647.5	505.6			535.4	505.7			541.2	505.6	
	719.5	577.5			607.3				613.4	577.5	
	791.7	649.5			679.4				685.3		
	863.6	721.6			751.6				757.4		
	935.6	793.6			823.6				829.5		
	1007.5	865.6			895.5						
					967.5						
OLATD1.3	287.4	145.1	163.1	OLAHX1.5		145	163.1	OLABA1.3		145	163.1
	359.3	217	235			217	235		253.1	217	235.2
	431.4	289.1	307.1			289.1	307		325.2	288.9	307.1
	503.4	361.2	379.3			361.1	379.1		397.2	361.2	379.1
	575.3	433.2	451.3		463.3	433.2	451.4		469.3	433.2	451.2
	647.4	505.6	523.4		535.4	505.7	523.4		541.3	505.5	523.2
	719.4	577.2	595.1		607.4	577.4	595.3		613.3	577.4	595.4
	791.55	649.4	667.3		679.3	649.3	667.3		685.3	649.2	667.3
	863.5	722.3	739.4		751.5	721.5	739.4		757.3	721.2	
	935.5	793.4	811.4		823.5	793.3			829.4	793.5	
	1007.5	864.5			895.5	865.4			901.4		
	1079.7	937.6			967.6				973.5		
	1151.7				1039.5				1045.4		
					1111.6						
					1183.7						

For the higher molar mass hexanol-initiated sample (OLAHX1.5), the mass spectrum revealed three distinct series (Figure 7B): (1) hexyl ester terminated chains and their transesterification byproducts (*m*/*z* = 463.3 to 1183.7, Δ_*m*/*z*_ = 72 Da), (2) cyclic oligomers (*m*/*z* = 145.05 to 865.4 Da), (3) acid-terminated chains (*m*/*z* = 163.1 to 739.4 Da). With increasing molar mass or chain length, the signal intensity of the ester-bound chains decreased, likely due to insufficient ionization, resulting in reduced detectability. In the higher molar mass region, a distinct series of peaks, (*m*/*z* = 831.5 to 1191.6 Da) was observed whose end-group structures remain unidentified. These peaks appeared consistent across the higher molar mass samples, irrespective of the initiator. Detailed analysis indicated a Δ_*m*/*z*_ = 8 Da shift relative to ester bound chains, while comparison with cyclic or acid-bound species showed variable mass differences, suggesting that the unassigned species likely originated from ester-terminated OLA chains.

**Figure 7:**
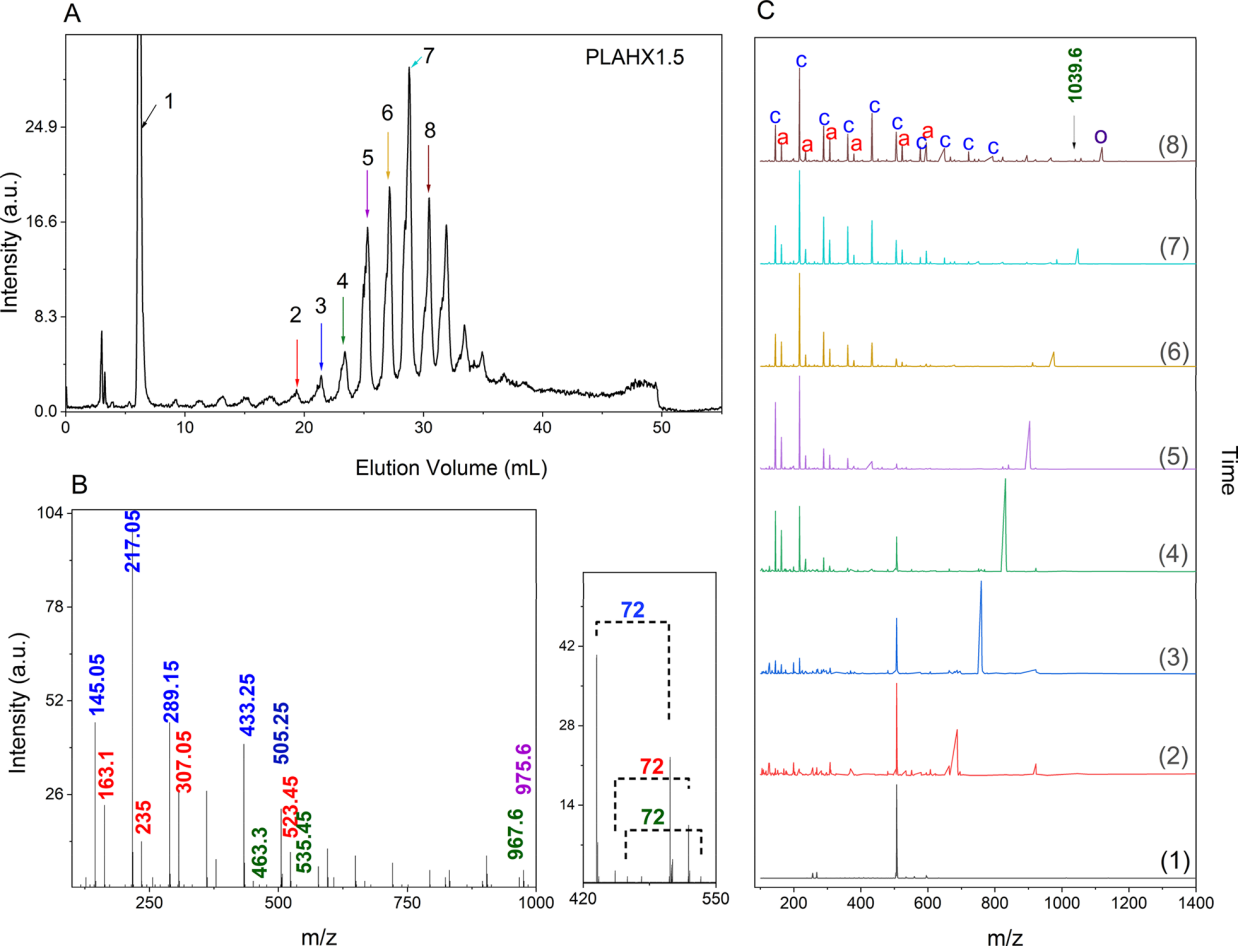
(A) Total ion chromatogram and (B) average spectra MS plot for OLA sample OLAHX1.5 based on final method step gradient from 0.4 vol% to 1.4 vol% EtOH in TCM in 55 min. Initiator bound species, cyclic species, acid-bound species, and unknown species highlighted in green, blue, red, and purple, respectively, (C) individual extracted mass spectra corresponding to peaks of OLAHX0.8 at: 6.1,19.3,21.4,23.4, 25.2, 27.1, 28.8, and 30.4 min. Cyclic species, acid bound species, and unknown species are highlighted with the marker -c-, -a-, and -o-, respectively.

As illustrated in Figure 7C, detailed peak analysis shows increasing contributions from cyclic and acid-terminated species across successive peaks. The peak at 30.49 min exemplifies the low ionization efficiency of ester-bound OLA chains, yielding signals barely distinguishable from background. In contrast, prominent signals at this retention time correspond to cyclic (’C’), acid-bound (’A’), and unknown (O’) species. The frequent coelution of cyclic and acid-terminated oligomers raises questions about the source of this heterogeneity. While acid species commonly form due to moisture during synthesis, thermal fragmentation in the ionization source may also contribute. Similar fragmentation has been reported in poly­(lactide-*co*-glycolide) copolymers by Masashi et al.^27^ This consideration is particularly relevant for the characterization of higher molar mass samples, which required elevated fragmentor voltages for detection. To enable a more accurate detailed end-group analysis, future studies should incorporate liquid chromatography under critical conditions (LCCC), which can better resolve minor end group differences and overcome the method’s sensitivity limitations. All unassigned OLA species, along with their tentative molecular formulas based on *m*/*z* analysis, are provided in supplementary Table S1.

The mass spectra of species eluting from overlapping peaks in the hexanol-initiated sample compared with those from the adjacent, nonoverlapping peaks in the benzyl alcohol-initiated sample as shown in Figure S9. The comparison reveals that the benzyl alcohol-initiated sample shows a notably higher intensity of the unknown species in the higher molar mass sample. This suggests that the presence and polarity of the unknown species potentially influenced by the benzyl end group contributes to differences in the shift in elution behavior, resulting in the peak separation observed in the overlaid elugrams shown in Figure 3.

Notably, the high-intensity unresolved peak at 49 min, observed in the bulk HPLC shown in Figure 6A and **7A,** is absent in LC-MS. This discrepancy, combined with (a) bulk analysis of OLAHX1.5 (Figure 1B), which detected OLA species up to *m*/*z* = 4000, and (b) the trend in Figure S5 showing increased intensity of the unresolved peak with greater sample mass, indicates that the missing peaks likely correspond to higher OLA species. Furthermore, these high mass chains are more prone to fragmentation during APCI ionization, suggesting the higher molar mass oligolactide species may originate from insource fragmentation of higher molar mass oligomer rather than being discrete oligomer populations. These observations underscore the limitations of LC–MS for higher molar mass species and highlight the need for complementary techniques such as MALDI-TOF MS, which are better suited for detecting intact high-mass oligomers, to achieve a comprehensive qualitative assessment of OLA.

## Conclusions

OLAs experienced a growing use in the biomedical field over the last years. Although their application necessitates a detailed analysis regarding end group heterogeneity and precise molecular size, the characterization of these oligomers has received little attention. We developed a novel HPLC method that enables effective separation of OLA samples using ethanol-modified chloroform on a normal-phase column. Besides varying the molar masses, OLAs having different end groups were used, which were synthesized by using the different initiators (1-hexanol, 1-tetradecanol, and benzyl alcohol) during ROP. This method shows excellent baseline separation and resolution for molar masses below 2000 g·mol^–1^ independent of the oligomer end group.

The hyphenation of this HPLC method with SEC in a 2D-LC system revealed that the peaks eluted in order of increasing molar mass. Coupling the HPLC with mass spectrometry enabled detailed compositional analysis, identifying cyclic OLAs and initiator-bound species with varying compositions. Extracted mass spectra of the separated peaks confirmed the observation that separation was governed by the degree of lactide incorporation. Coelutions seen in both the 2D-LC and peak-level analysis emphasized the method’s focus toward separating initiator-bound OLAs by lactide incorporation rather than end-group functionality. These findings highlight the molecular heterogeneity intrinsic to OLA samples synthesized via ring-opening polymerization and reinforce that mass spectrometry and chromatography go hand-in-hand in allowing for its identification.

## Supplementary Material



## Data Availability

The experimental data of this study are published on the open repository Zenodo at https://doi.org/110.5281/zenodo.17987223.

## Notes

Synthesis; HPLC method development; method for 2D-LC; LC-MS data (PDF)
