# Supplementary material for: Comprehensive Characterization of Oligolactide Architecture by Multidimensional Chromatography and Liquid Chromatography–Mass Spectrometry
Source: ACS Omega. 2026 Jan 16;11(4):5295–305. doi: 10.1021/acsomega.5c08063 (PMC12878347; doi:10.1021/acsomega.5c08063)
Supplement: Supplementary file 1 [file ao5c08063_si_001.pdf]

Supplementary Information

Comprehensive Characterization of Oligolactide Architecture by Multidimensional Chromatography and LC–MS

Amit Lal[a], Paul Eselem Bungu[a], Karola Luetzow*[a], Sarah Kirchhecker[a], Regine Apostel[a], Olaf Lettau[a], Monique Hannemann[a], Harald Pasch[b], Francesca M. Toma[a,c]*

[a]Institute of Functional Materials for Sustainability, Helmholtz-Zentrum Hereon, Kantstr. 55, 14513 Teltow, Germany

[b]Institute of Active Polymers, Helmholtz-Zentrum Hereon, Kantstr. 55, 14513 Teltow, Germany

[c]Faculty of Mechanical and Civil Engineering, Helmut Schmidt University, 22043 Hamburg, Germany

Supplementary Information

Ring-opening polymerization of L-lactide with different target molar masses and different initiators:

0.75 g (5.2 mmol) L-lactide, 10 µL (30.9 µmol) Sn(Oct)2, and the initiator (1-hexanol, 1-tetradecanol or benzyl alcohol) were mixed under N2 atmosphere and stirred at 120 ˚C for 1.5 h. The materials were used without further purification. The amount of initiator was varied according to the targeted molar mass as listed below:

For targeted molar mass 500 Da: 565 mg (2.65 mmol) 1-tetradecanol (OLATD0.7) or 198.8 µL (1.91 mmol) benzyl alcohol (OLABA0.7) or 237 µL (1.89 mmol) 1-hexanol (OLAHX0.8)

For targeted molar mass 1000 Da: 202.7 mg (0.95 mmol) 1-tetradecanol (OLATD1.3) or 89.5 µL (0.86 mmol) benzyl alcohol (OLABA1.3) or 105 µL (0.84 mmol) 1-hexanol (OLAHX1.0, OLAHX1.5)

HPLC method development

Solvent selection

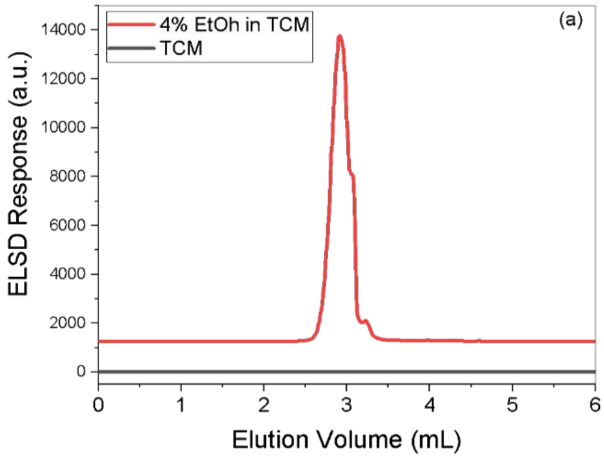

Figure S 1. Comparison of elution profiles of OLA1.5, a commercial GPC standard, obtained by 10 min isocratic elution with TCM vs 4 vol.% EtOH in TCM; Nucleosil-OH, at 35˚C.

The adsorbing effect at 100 % TCM is illustrated by the complete adsorption of OLA 1.5 in Figure S1. For the desorbing eluent, ethanol-modified TCM containing 4 vol.% absolute ethanol,

referred to here as TCM-OH, was used. The effectiveness of TCM-OH as a promising desorption-promoting eluent is demonstrated by the complete desorption of OLA1.5, with a sample recovery of more than 95 % of the injected sample, as calculated from the highlighted peak areas in Figure S2.

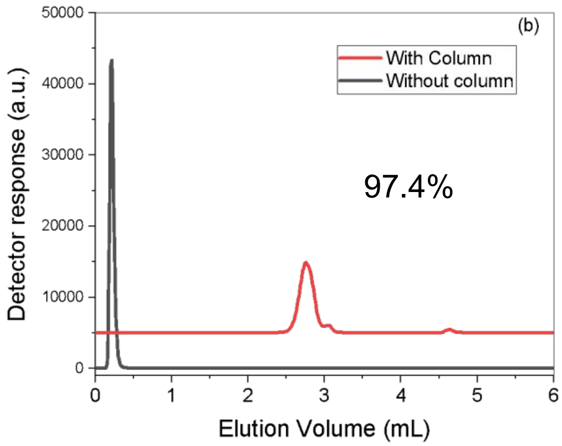

Figure S 2. Comparison of area under elution curve of OLA1.5 obtained by 6 min isocratic elution with column (-) and without column (-) for 4 vol.% EtOH in TCM; Nucleosil-OH, at 35˚C.

Solvent effect

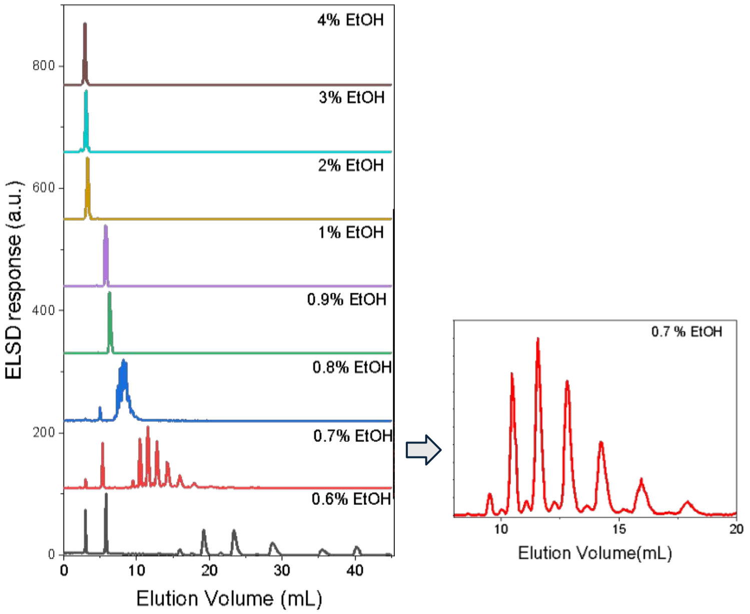

Figure S 3. Comparison of elution profiles of OLA1.5 obtained by isocratic elution with varying percentages of EtOH in TCM (0.6 vol.%, 0.7 vol.%, 0.8 vol.%, 0.9 vol.%, 1 vol.%, 2 vol.%, 3 vol.%, 4 vol.%); Magnified elution profile of OLA1.5 showing two different species obtained by isocratic elution with 0.7 vol.% EtOH in TCM;

The initial steps for an isocratic elution that was conducted with varying eluent compositions to identify the optimal composition for detecting the separation of OLA1.5 molecules, as shown in Figure 4.2A. The composition of TCM and TCM-OH was systematically adjusted using the pump to achieve TCM-OH composition with increasing EtOH content. Interestingly, even a small change of 0.1 vol.% ethanol content significantly affects the elution profiles of OLA 1.5, particularly evident in the chromatograms obtained with TCM-OH containing ethanol content ranging between 0.6 to 1 vol.%. Decreasing the ethanol composition from 1 to 0.6 vol.% enhanced the separation of OLA 1.5 K molecules into multiple peaks.

Temperature effect

The effect of temperature on the adsorption and desorption of the oligolactide molecules was equally investigated at eluent compositions containing 0.7 vol.%, 0.8 vol.%, and 0.9 vol.% ethanol content in TCM. Contrary to the norm, where an increase in the column temperature often leads to a decrease in elution volume, oligolactide chains show an inverse temperature effect exhibiting an increase in adsorption as the column temperature is increased from 30 to 55˚C. This unique retention characteristic is common for polymers with an amphiphilic nature, allowing them to interact with both the non-polar column substrates and polar mobile phase, leading to an entropy gain from releasing solvated molecules. Given that OLA shares a similar amphiphilic nature, it is plausible that OLA may exhibit an analogous retention behavior in chromatographic separations at higher temperatures.

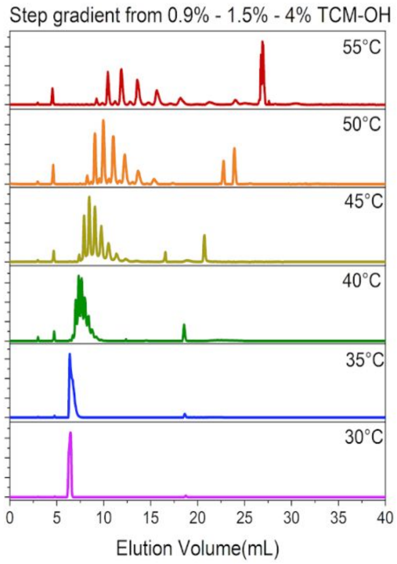

Figure S 4. Comparison of elution profiles of OLA1.5 at varying temperatures (35˚C, 40˚C, 45˚C, 50˚C, 55˚C) from 0.9 vol.% to 4 vol.% EtOH in TCM; Nucleosil-OH.

Unresolved peak

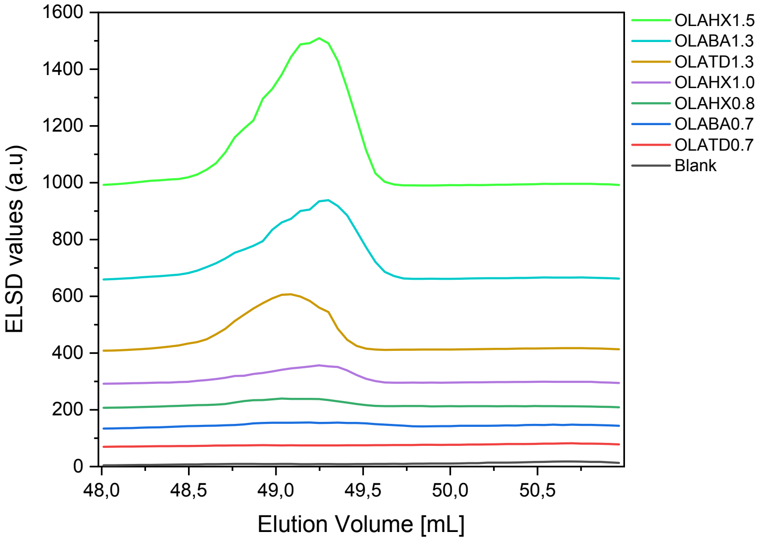

Figure S 5. Enhanced plot to show intensity of unresolved peak with increase in molar mass of samples.

Modified method for 2D-LC

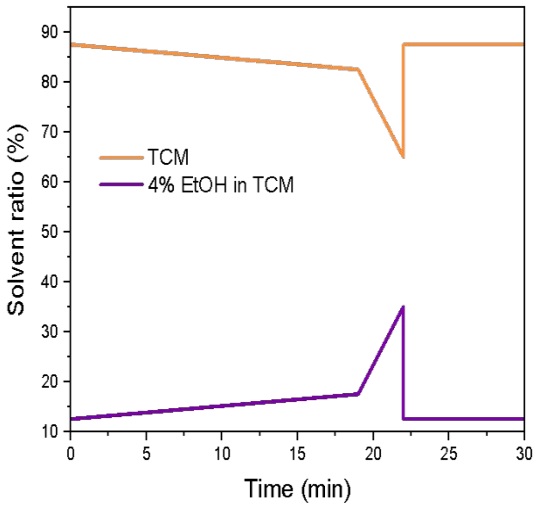

Figure S 6. Modified gradient profile from 0.5 vol.% to 1.4 vol.% EtOH in TCM for 30 mins.

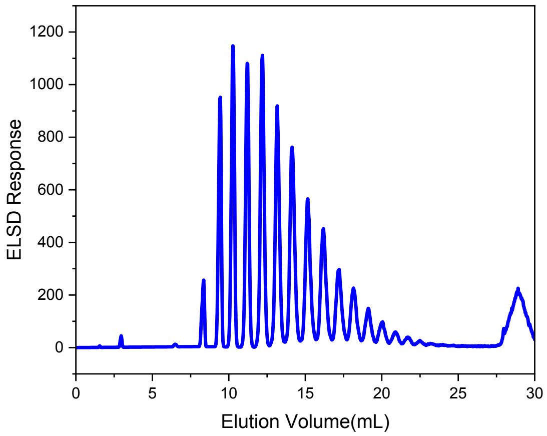

Figure S 7. Elution profile of OLABA0.7 obtained by applied 30 min gradient; stationary phase Nucleosil-OH, temperature 30˚C.

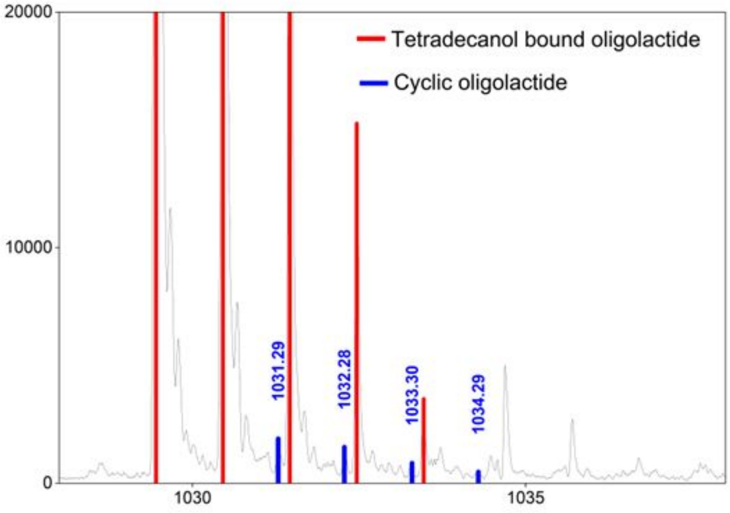

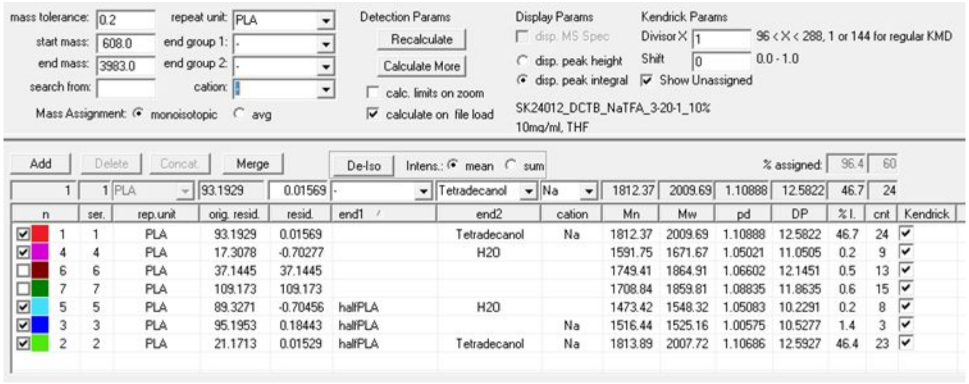

n	ser.	rep.unit	orig. resid.	resid.	end1	end2	cation	Mn	Mw	pd	DP	% I.	cnt	Kendrick
1	1	PLA	93.1929	0.01569		Tetradecanol	Na	1812.37	2009.69	1.10888	12.5822	46.7	24	✓
4	4	PLA	17.3078	-0.70277		H20		1591.75	1671.67	1.05021	11.0505	0.2	9	✓
6	6	PLA	37.1445	37.1445				1749.41	1864.91	1.06602	12.1451	0.5	13	✓
7	7	PLA	109.173	109.173				1708.84	1859.81	1.08835	11.8635	0.6	15	✓
5	5	PLA	89.3271	-0.70456	halfPLA	H20		1473.42	1548.32	1.05083	10.2291	0.2	8	✓
3	3	PLA	95.1953	0.18443	halfPLA		Na	1516.44	1525.16	1.00575	10.5277	1.4	3	✓
2	2	PLA	21.1713	0.01529	halfPLA	Tetradecanol	Na	1813.89	2007.72	1.10686	12.5927	46.4	23	✓

Figure S 8: cyclic species observed on the Maldi, evident from the fitting isotope pattern and ~2% intensity on the Polytools.

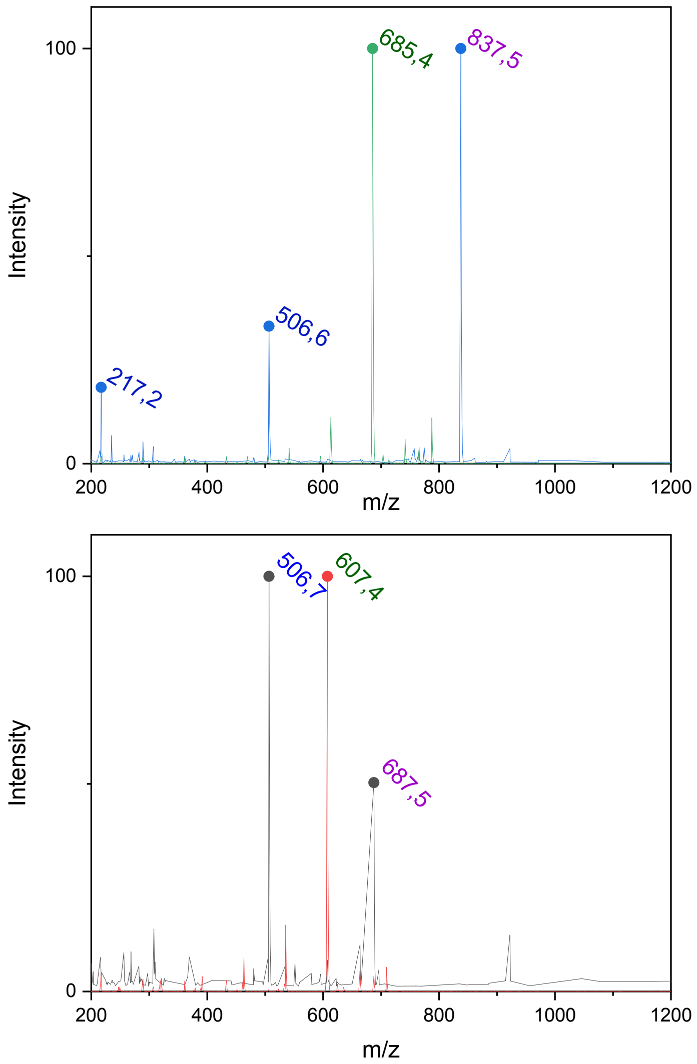

Figure S 9: Mass spectra for the comparison of eluting species in non-overlapping peaks of benzyl alcohol-initiated samples (OLABA0.7 at time 23 mins (●) and OLABA1.3 at 24 mins (●)); and overlapping peaks of hexanol initiated samples OLAHX0.8 (●) and OLAHX1.5(●) at 19 mins; with hexanol-initiated oligolactide series highlighted in green, cyclic oligolactide species highlighted in blue and unknown oligolactide species highlighted in purple.

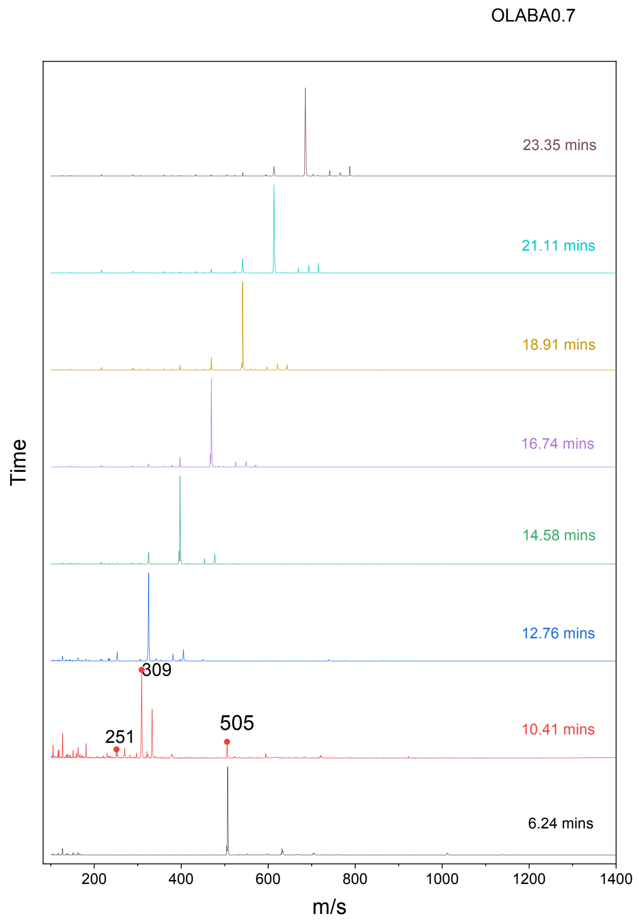

Figure S 10: Extacted mass spectrum of benzyl alcohol sample (OLABA0.7) showing cyclic species at minute 10.41

LC-MS

Table S1. Identification of oligolactide species in samples with corresponding molecular formulas based LC-MS coupling using APCI as ionization source. Complete list of all species found.

**Initiator**	**Tetradecanol**			**Hexanol**			**Benzyl Alcohol**	
End group	Unknown Series (Initiator bound +31m/z)	Unknown Series (Initiator bound end group series +8m/z)	End group	Unknown Series (Initiator bound +31m/z)	Unknown Series (Initiator bound end group series +8m/z)	End group	Unknown Series (Initiator bound +31m/z)	Unknown Series (Initiator bound end group series +8m/z)
Sample	$[M+H]^+$		Sample	$[M+H]^+$		Sample	$[M+H]^+$	
OLATD0.7			OLAHX0.8	637.6<br>709.4<br>781.3<br>853.7<br>925.65<br>997.65		OLABA0.7	571.5<br>643.5<br>715.6<br>787.35<br>859.5<br>931.7	
OLATD1.3		655.5<br>727.6<br>799.7<br>871.75	OLAHX1.5		687.5<br>759.5<br>831.6<br>903.6<br>975.6<br>1047.7<br>1119.6<br>1191.6	OLABA1.3	549.4<br>621.4<br>693.4<br>765.5<br>837.4	693.4<br>765.4<br>837.4<br>909.5<br>981.7<br>10536
